# Characteristic distribution of the total and individual item scores on the Kessler Screening Scale for Psychological Distress (K6) in US adults

**DOI:** 10.1186/s12888-017-1449-1

**Published:** 2017-08-07

**Authors:** Shinichiro Tomitaka, Yohei Kawasaki, Kazuki Ide, Maiko Akutagawa, Hiroshi Yamada, Yutaka Ono, Toshiaki A. Furukawa

**Affiliations:** 1 Department of Mental Health, Panasonic Health Center, Landic building 3F, Nishishinbashi 3-8-3, Minato-ku, Tokyo, 105-0003 Japan; 2 Department of Drug Evaluation and Informatics, School of Pharmaceutical Sciences, University of Shizuoka, 52-1 Yada, Suruga-ku, Shizuoka, 422-8526 Japan; 3 Department of Health Promotion and Human Behavior, Department of Clinical Epidemiology, Kyoto University Graduate School of Medicine/School of Public Health, Yoshida Konoe-cho, Sakyo-ku, Kyoto, 606-8501 Japan; 4 Department of Pharmacoepidemiology, Graduate School of Medicine and Public Health, Kyoto University, Yoshida Konoe-cho, Sakyo-ku, Kyoto, 606-8501 Japan; 5 Center for the Promotion of Interdisciplinary Education and Research, Kyoto University, Yoshida-honmachi, Sakyo-ku, Kyoto, 606-8501 Japan; 6 Center for the Development of Cognitive Behavior Therapy Training, Shirogane-cho 1-13, Shinjuku-ku, Tokyo, 162-0816 Japan

**Keywords:** Depressive symptom, Kessler Screening Scale for Psychological distress, Item response, National Survey of midlife development, Exponential distribution, Depressive symptom measure

## Abstract

**Background:**

The distributional pattern of total scores on depression screening scales in the general population has not been well studied. Recent studies suggest that the total scores on depression screening scales follow an exponential pattern, with the exception of the lower end of the distribution. To further investigate the findings, we determined the distributions of the total and individual item scores on the Kessler Screening Scale for Psychological Distress (K6).

**Methods:**

Data were obtained from the National Survey of Midlife Development in the United States. Participants comprised 6,223 individuals between the ages of 25 and 74. The distributions of the total and individual item scores in various combinations were investigated with histograms and regression analysis.

**Results:**

Irrespective of the combination of items, the total and individual item scores followed an exponential pattern except at the lower scores. The estimated rate parameters of regression analysis were similar among distributions with the same number of chosen items. At the lower scores, the distributional patterns of total scores varied according to the ratio of “a little” to “none” for each item response.

**Conclusions:**

The present results have the potential to estimate the distribution of depressive symptoms in the general population. While the degree of depressive symptoms varies from individual to individual, an entire population may show a certain mathematical distribution.

## Background

Depression is a common mental disorder that affects about 350 million people worldwide [1, 2]. Because the degree of depressive symptoms is fundamental to a diagnosis of clinical depression, the severity distribution of depressive symptoms in the general population is a major area of interest within the field of psychiatry [3, 4]. The distribution of depressive symptoms is significant as well because statistical hypothesis tests and statistical estimators are derived from statistical models, which are assumed to adequately approximate the empirical distribution [5].

Population studies of depressive symptoms have been conducted using parametric statistics, factor analysis, and item response theory. These methods are called confirmatory data analysis (CDA), which presupposes a statistical model, tests a hypothesis, and estimates parameters [6]. On the other hand, exploratory data analysis (EDA) is an inductive approach designed to reveal characteristics and patterns in the data, often with visual methods [6, 7]. Both inductive and deductive approaches are necessary for data analysis. However, whereas CDA has been performed by a large number of researchers, exploratory data analysis has not drawn attention in the field of psychiatry. For example, little work has been done to visualize the distributions of depressive symptom scores in the general population to understand their characteristics patterns.

Visualizations are central to EDA because the rich information they provide is unrivaled in its ability to detect data patterns [8, 9]. Recently, through visualizations with histograms, several large sample studies have demonstrated that total scores on depression screening scales in the general population approximate exponential distributions, with the exception of the lower end of the distribution. An analysis of data from almost 10,000 respondents to the British National Household Psychiatric Morbidity Survey has revealed that an exponential model best fits the total score data on the Revised Clinical Interview Schedule (CIS-R), except at the lowest symptom counts [10]. Analyzing almost 25,000 respondents to the Japanese Active Survey of Health and Welfare, we have similarly reported that the distribution of total scores on the Center for Epidemiologic Studies Depression Scale (CES-D) approximates an exponential pattern, with the exception of the lower end of the distribution [11]. These findings were consistently confirmed in a sample of about 7600 Japanese employees using the CES-D [12].

To verify the findings, we determined the total score distributions of the Kessler Screening Scale for Psychological Distress (K6) in representative US studies. The K6 assesses the degree of psychological distress and identifies respondents with a diagnosable mental illness [13]. Although the K6 was designed to measure psychological distress [14], the six items of the K6 (depressed mood, motor agitation, fatigue, worthless guilt, and worry) may be grouped as depressive symptoms. The K6 is a reliable and valid tool to screen for depressive symptoms and mood disorders in epidemiological survey [15, 16]. The distributional pattern of the total scores has also been repeated in two analyses of K6 data from the National Survey of Midlife Development (MIDUS) and the National Health Interview Survey (NHIS) [17, in submission].

Furthermore, we investigated the total scores of the Patient Health Questionnaire (PHQ) in a representative US survey (in submission). The PHQ is a globally used self-rating screening scale for depression [18, 19]. In an analysis of data from the eight-item version of the Patient Health Questionnaire (PHQ-8) of the Behavioral Risk Factor Surveillance Survey (BRFSS) in the United States, we confirmed that total scores followed an exponential pattern, except for the lowest symptom counts [in submission].

Taken together, the distributional pattern of the total scores concurs with results of the national surveys utilizing the CIS-R, CES-D, K6, and PHQ-8. If the distributional patterns of the total scores on depression screening scales in the general population is established, a new perspective will be presented on how depressive symptoms are distributed in the general population. Furthermore, if the pattern of the empirical distribution follows a non-normal distribution, the statistical model of normal distributed depressive symptom scores, which is presupposed in parametric statistics and item response theory, requires reconsideration. To confirm reproducibility of these findings, further studies are necessary.

The results of previous studies suggest that regardless of the combination of items, total scores on depression screening scales follow an exponential pattern, with the exception of the lower end of the distribution. Thus, in our previous studies, we analyzed the distributions of the total scores of the chosen items on the CES-D in various combinations and found that for any combination of items, the total scores of the selected items approximated an exponential pattern, with the exception of the lowest scores [20]. Furthermore, we observed that (1) the total score distributions with the same number of chosen items exhibited similar estimated rate parameters; (2) the estimated rate parameters increased in proportion to the number of chosen items; and (3) the ratio of “none” to “some” of item responses contributed to the non-exponential pattern of total score distributions at the lowest scores [20]. These findings suggest that the distributional patterns of the total scores are dependent on the item responses of such items. However, there are few studies that have investigated whether the aforementioned findings are reproduced for other scales.

The MIDUS is a collaborative investigation of midlife development in the areas of physical health and psychological well-being [21]. The K6 has been included as part of the MIDUS questionnaires. All MIDUS datasets and documentation are archived at the Interuniversity Consortium for Political and Social Research (ICPSR) repository and are publicly available [21]. Since data of MIDUS are large sample sizes with limited selection bias, they are suitable to confirm the previous findings. The present study used data from the MIDUS to verify aforementioned findings.

The aim of the present study was threefold: (1) to confirm that, regardless of the combination of chosen items, the total scores of chosen items on the K6 show an exponential curve, with the exception of the lower end of the distribution; (2) to investigate the relationship between the number of chosen items and the estimated rate parameters of the exponential model; and (3) to examine how the ratio of “a little” to “none” in item responses contributes to the patterns of the total scores at the lower end of the distribution.

## Methods

### Data set

Data were obtained from the first wave of MIDUS (MIDUS 1) [21, 22]. The MacArthur Foundation Research Network on Successful Midlife Development conducted the MIDUS 1 between 1995 and 1996. The MIDUS 1 comprised a nationally representative sample of adults between the ages of 25 and 74 in the United States. The average age of the sample was 46.4 years (SD = 13). Non-English speaking and institutionalized individuals were excluded. The MIDUS 1 sample consisted of 7108 participants (male: *n* = 3395) including four subsamples: the main sample from a national random-digit-dialing (RDD) procedure, oversamples from five US areas, siblings of participants from the RDD sample, and a national sample of twin pairs. A detailed description of response rates and sociodemographic characteristics has been published elsewhere [22]. The institutional review board at each participating site approved the MIDUS, and all participants provided informed consent.

### Ethics statement

This study used de-identified data available to the public. The author’s institutional review board did not consider the secondary analysis of publicly available data as human subjects research.

### Measures

The self-administered questionnaire of the MIDUS 1 included the K6 items. The K6 consists of six items asking about the frequency of feeling (1) sad, (2) nervous, (3) restless or fidgety, (4) hopeless, (5) that everything was an effort, and (6) worthless in the last 30 days (Table 1). All items are scored on a 5-point scale from 0 (none of the time) to 4 (all of the time). A total item score ranges from 0 to 24. One of the K6 items used in the MIDUS questionnaires is expressed as follows: “How much of the time did you feel so sad that nothing could cheer you up?” This question slightly differs from that generally used in the standard K6: “How often did you feel so depressed that nothing could cheer you up?’ [14].

**Table 1:** Item responses of respondents (*N* = 6223)

Item	Response, n (%)					Ratio of “a little” to “none”	Ratio of “some” to “a little”	Rank order of the ratio of “a little” to “none”
	None	A little	Some	Most	All			
1 Sad	4383 (70.4)	1260 (20.2)	462 (7.4)	97 (1.6)	21 (0.3)	0.29	0.37	3
2 Nervous	2718 (43.7)	2253 (36.2)	1011 (16.2)	199 (3.2)	42 (0.7)	0.83	0.45	6
3 Restless	2951 (47.4)	2042 (32.8)	997 (16.0)	184 (3.0)	49 (0.8)	0.69	0.49	5
4 Hopeless	5000 (80.3)	768 (12.3)	330 (5.3)	91 (1.5)	34 (0.5)	0.15	0.43	2
5 Effort	3632 (58.4)	1664 (26.7)	637 (10.2)	214 (3.4)	76 (1.2)	0.46	0.38	4
6 Worthless	5004 (80.4)	765 (12.3)	326 (5.2)	89 (1.4)	39 (0.6)	0.15	0.43	1
Average	3948 (63.4)	1459 (23.4)	627 (10.1)	146 (2.3)	44 (0.7)	0.43	0.42	

### Analysis procedure

We excluded 885 respondents (12.5%) from the present analysis because they did not respond to all six items. The final sample consisted of 6223 respondents, including 2975, 647, 858, and 1743 respondents from the national RDD sample, siblings, twin pairs, and oversamples, respectively.

First, item response rates were calculated for all 6 items. As the previous study demonstrated, the ratio of “a little” to “none” contributes to the distributional pattern at the lower end of the distribution [20]. According to the previous study, while the total scores of items with high values of the ratio of “a little” to “none” are expected to exhibit lower frequencies than predicted from the exponential regression curve, the total scores of items with low values of the ratio of “a little” to “none” are expected to exhibit higher frequencies than predicted from the regression curve. On the other hand, the ratios of “some” to “a little” were similar among all 6 items [20]. Therefore, the ratios of “a little” to “none,” and “some” to “a little” were calculated for all 6 items. All 6 items were ranked according to the ratio of “a little” to “none” in ascending order. Thereafter, patterns of item responses were analyzed with graphical analysis.

The distributions of the total scores of the chosen K6 items for various combinations were investigated using histograms and regression analysis. As an exponential curve exhibits a linear pattern with a semi-logarithmic scale, a semi-logarithmic scale allows us to identify the range of an exponential pattern. Regression analysis was used to estimate the relationship between the sum of the K6 and the frequencies. The total score distributions of 2 items, 3 items, 4 items, and 5 items were analyzed in various combinations. According to the ranking of the ratio of “a little” to “none,” the 6 items of the K6 were organized into groups. The exponential regression analysis was conducted using the least square method. Descriptive statistics and frequency distribution curves were calculated using SAS JMP (Version 11 for Windows).

## Results

### Item responses for K6 items and the distribution of total item scores

Item responses to the K6 are shown in Table 1. The item responses for the 6 items showed a certain pattern, with the highest response rate for “none,” a decreasing response rate with the increasing item score, and the lowest response rate for “all.” While the ratio of “a little” to “none” varied by item (mean ± SD = 0.43 ± 0.28), the ratio of “some” to “a little” was stable for all items (mean ± SD = 0.42 ± 0.04). All 6 items were ranked according to the ratio of “a little” to “none” in ascending order.

**Fig. 1:**
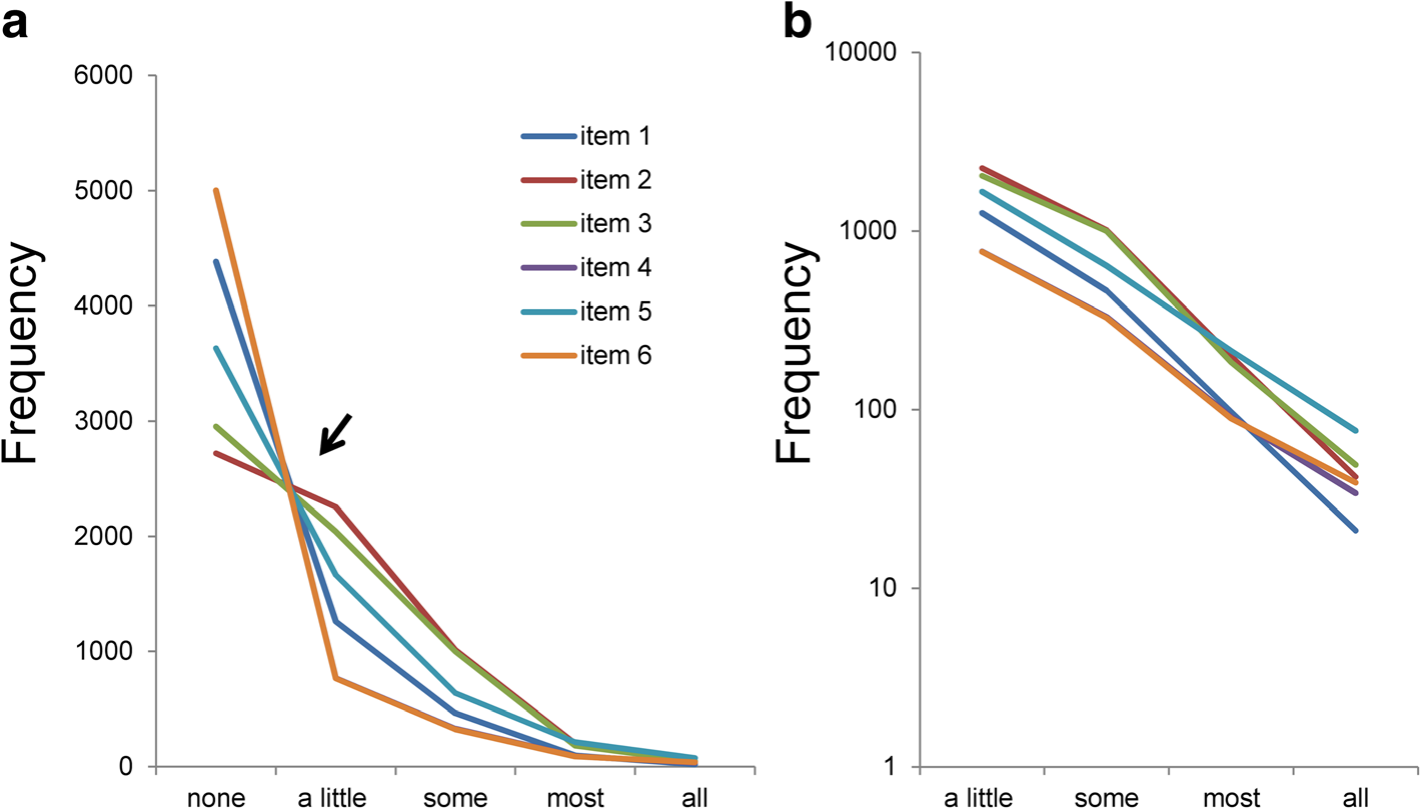
Item responses for 6 items. The item responses for 6 items are presented for normal (**a**) and semi-logarithmic (**b**) scales. **a** The item responses for each of the 6 items showed a common pattern. The lines for the 6 items crossed between “none” and “a little,” whereas the same lines exhibited a right-skewed pattern between “a little” and “all.” The line of item 4 is hiding behind that of item 6. The responses of Item 4 and 6 are very similar (Table 1). **b** Using a semi-logarithmic scale, the item responses for the 6 items showed a linear pattern between “a little” and “all”

For the pattern analysis of item responses, all response frequencies were plotted on a single graph. As presented in Fig. 1a, the item response frequencies demonstrated a certain pattern. As pointed by the arrow (Fig. 1a), the lines for the 6 items intersected at a single point between “none” and “a little,” whereas the same lines decreased regularly and converged at one point. As verified in our previous studies, if the ratios of “some” to “a little,” “most” to “some,” and “all” to “most” are similar among all items, lines for item responses intersect at a single point between “none” and “a little” as expected [23].

Using a semi-logarithmic scale (Fig. 1b), the item responses of the 6 items approximated a linear pattern between “a little” and “all of the time.” The slopes of the lines for the 6 items were similar between “a little” and “all.” The extent of parallelism of the 6 items represents the similarity of the ratios of “some” to “a little,” “most” to “some,” and “all” to “most” among the 6 items. Exponential regression analysis was performed on the data from 1 to 5 points (“a little” to “all”) for item 1 (y = 5909e^-1.384x^, R^2^ = 0.99), item 2 (y = 11054e^-1.357x^, R^2^ = 0.98), item 3 (y = 9211e^-1.288x^, R^2^ = 0.98), item 4 (y = 2392e^-1.064x^, R^2^ = 0.99), item 5 (y = 4817e^-1.035x^, R^2^ = 0.99), and item 6 (y = 2211e^-1.023x^, R^2^ = 0.99). The predictor variable (x) was the item score, the predicted variable (y) was the frequency of respondents, and R^2^ was the coefficient of determination. The analysis showed high R^2^ values, indicating that the exponential models provided a good fit to the data with similar regression coefficients (−1.023 to −1.384).

**Fig. 2:**
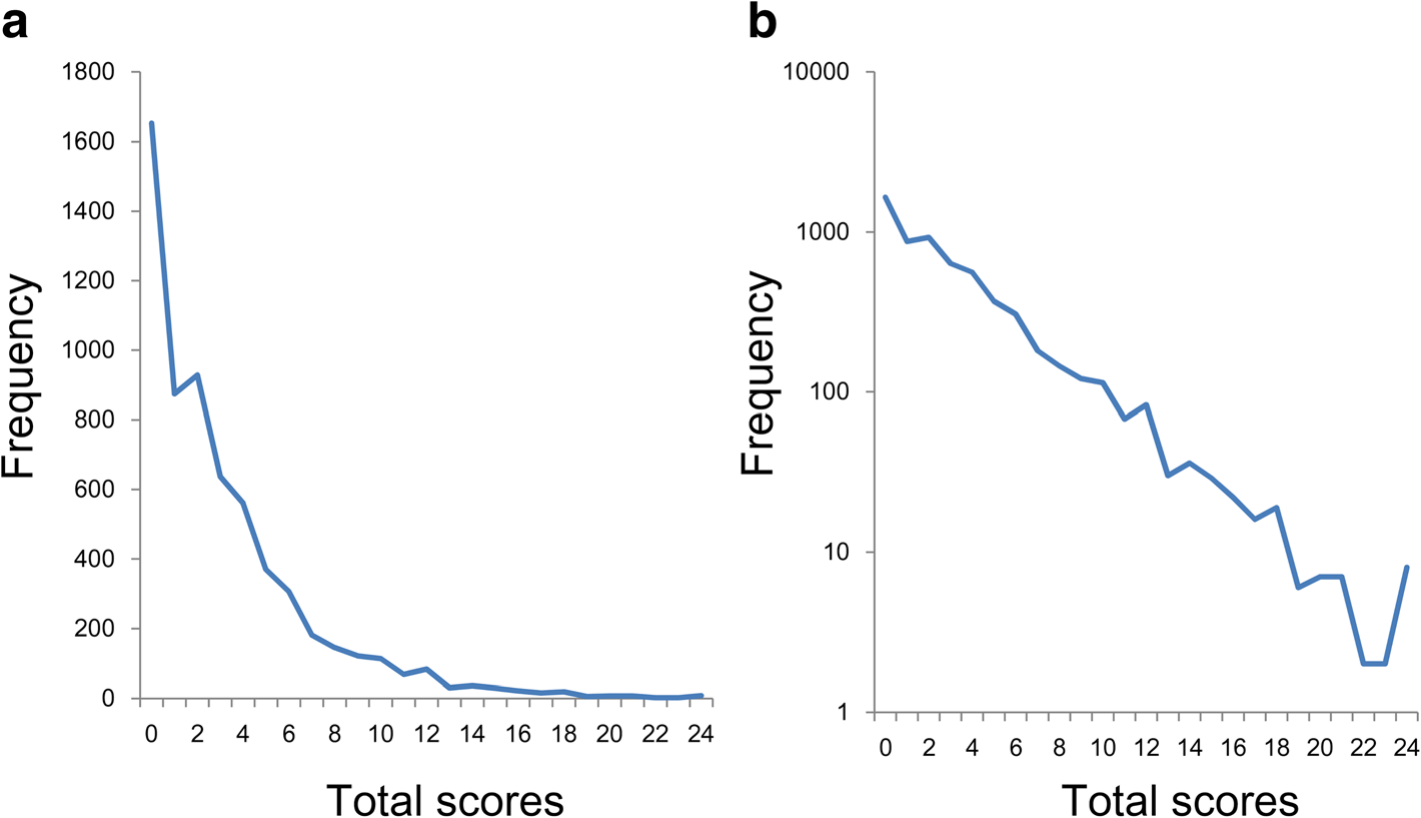
Distributions of the total scores of the K6. **a** The distribution of the total score was right-skewed on a normal scale. **b** The distribution of the total score showed a linear pattern on a semi-logarithmic scale

As shown in Fig. 2, we examined the total score distribution of the 6 items. The distribution was positively skewed (Fig. 2a). Using a semi-logarithmic scale (Fig. 2b), the total scores exhibited a linear pattern for almost the whole extent of the distribution. Of note, the total scores fluctuated more with the increase of total scores, consistent with previous studies [11, 17]. The fluctuation may be due to the small sample sizes at the highest scores. The possibility that the small sample sizes caused the fluctuations is supported by the fact that the frequencies at the higher scores were very low (less than 10).

Exponential regression analysis was performed on the total score data (y = 1787.7e^-0.26x^, R^2^ = 0.97). The predictor variable (x) was the total score, and the predicted variable (y) was the frequency of participants. The analysis showed a high R^2^ value, indicating that total scores showed a good fit to an exponential distribution.

### Distributions of the sum of 5 items

Next, the total score distributions of 5 item scores were analyzed. Based on the ranking order of the ratio of “a little” to “none,” two groups of items were chosen: low ratio group (items 1, 3, 4, 5, and 6) and high ratio group (items 1, 2, 3, 4, and 5). The low ratio group comprised items from first to fifth place in the ranking of the ratio of “a little” to “none” in ascending order, and the high ratio group consisted of items ranked second to sixth. The average of the ratio of “a little” to “none” was 0.35 and 0.48 for the low ratio group and high ratio group, respectively.

**Fig. 3:**
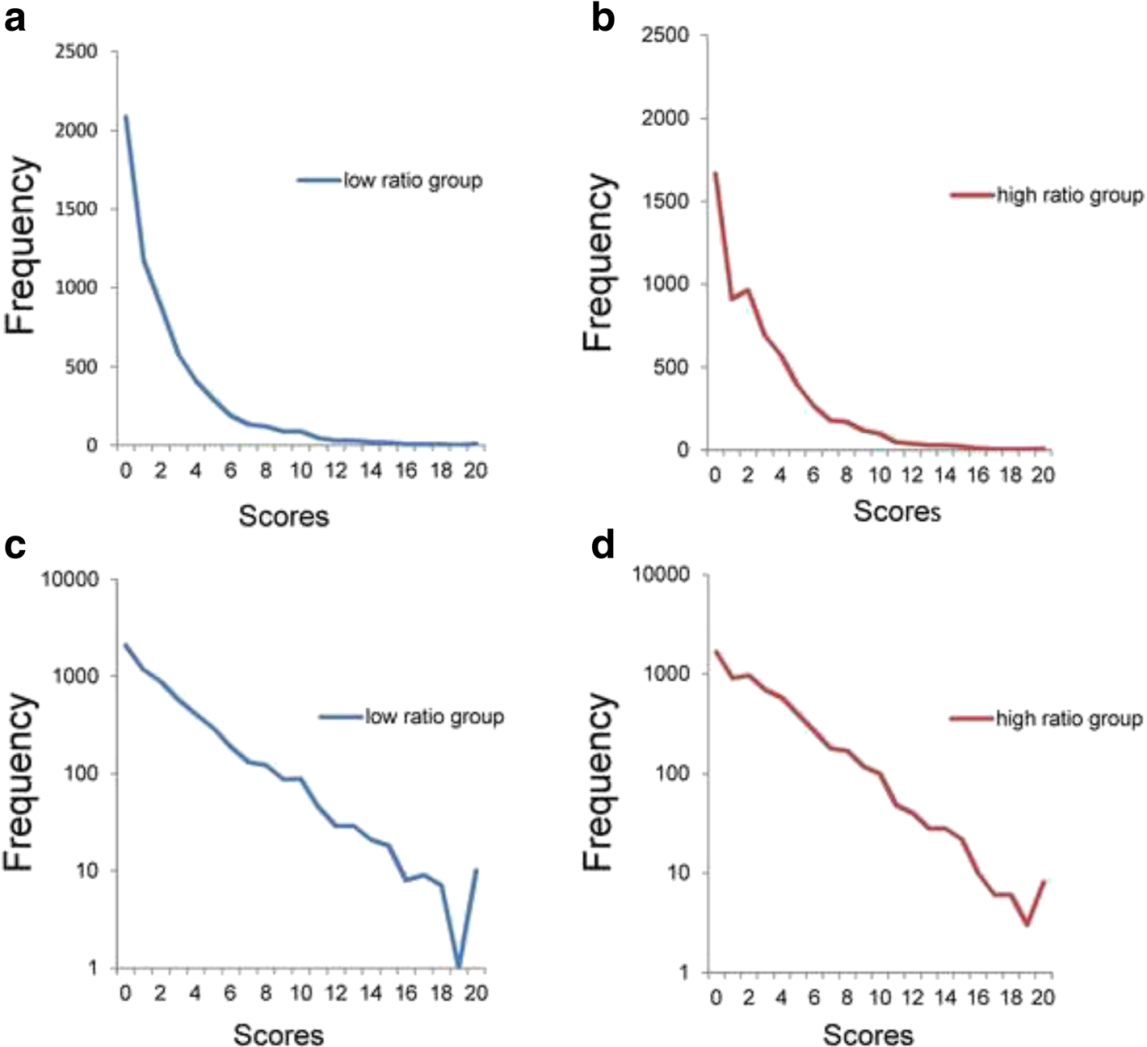
Distributions of the total scores of the 5 items. **a** Low ratio group (items 1, 3, 4, 5, and 6) and (**b**) high ratio group (items 1, 2, 3, 4, and 5) on a normal scale. **c** Low ratio group and (**d**) high ratio group on a semi-logarithmic scale. The distributions of the total scores of 5 item scores for the two groups were commonly right-skewed (**a**, **b**). Using semi-logarithmic scales, the two groups showed linear patterns with similar gradients (**c**, **d**)

The distributions for the two groups are depicted in Fig. 3a (low ratio group) and Fig. 3b (high ratio group). Both of the distributions were positively skewed. Using a semi-logarithmic scale (Fig. 3c and d), the two groups exhibited linear patterns with similar slopes from 0 to 20 points, indicating that the two distributions exhibited an exponential pattern, with similar regression coefficients. Consistent with the total scores of the 6 items (Fig. 2b), the distribution fluctuated at the highest scores (Fig. 3c and d).

Exponential regression analysis was perfrmed on data of the low ratio group from 0 to 24 points (y = 2059.7e^-0.311x^, R^2^ = 0.95) and high ratio group from 0 to 24 points (y = 2275e^-0.306x^, R^2^ = 0.98). In line with semi-logarithmic scale findings, the regression analysis showed high coefficients of determination (R^2^, 0.95 to 0.98) with similar rate parameters (−0.306 to −0.311).

### Distributions of the sum of 4 items

Based on the ranking order of the ratio of “a little” to “none,” three groups were chosen: low ratio group (items 1, 4, 5, and 6), middle ratio group (items 1, 3, 4, and 5), and high ratio group (items 1, 2, 3, and 5). The low ratio group comprised items from the first to the fourth rank order of the ratio of “a little” to “none,” the middle ratio group comprised items from the second to fifth, and high ratio group comprised items from the third to sixth. The averages of the ratio of “a little” to “none” were 0.26, 0.40, and 0.57 for the low ratio group, middle ratio group, and high ratio group, respectively.

**Fig. 4:**
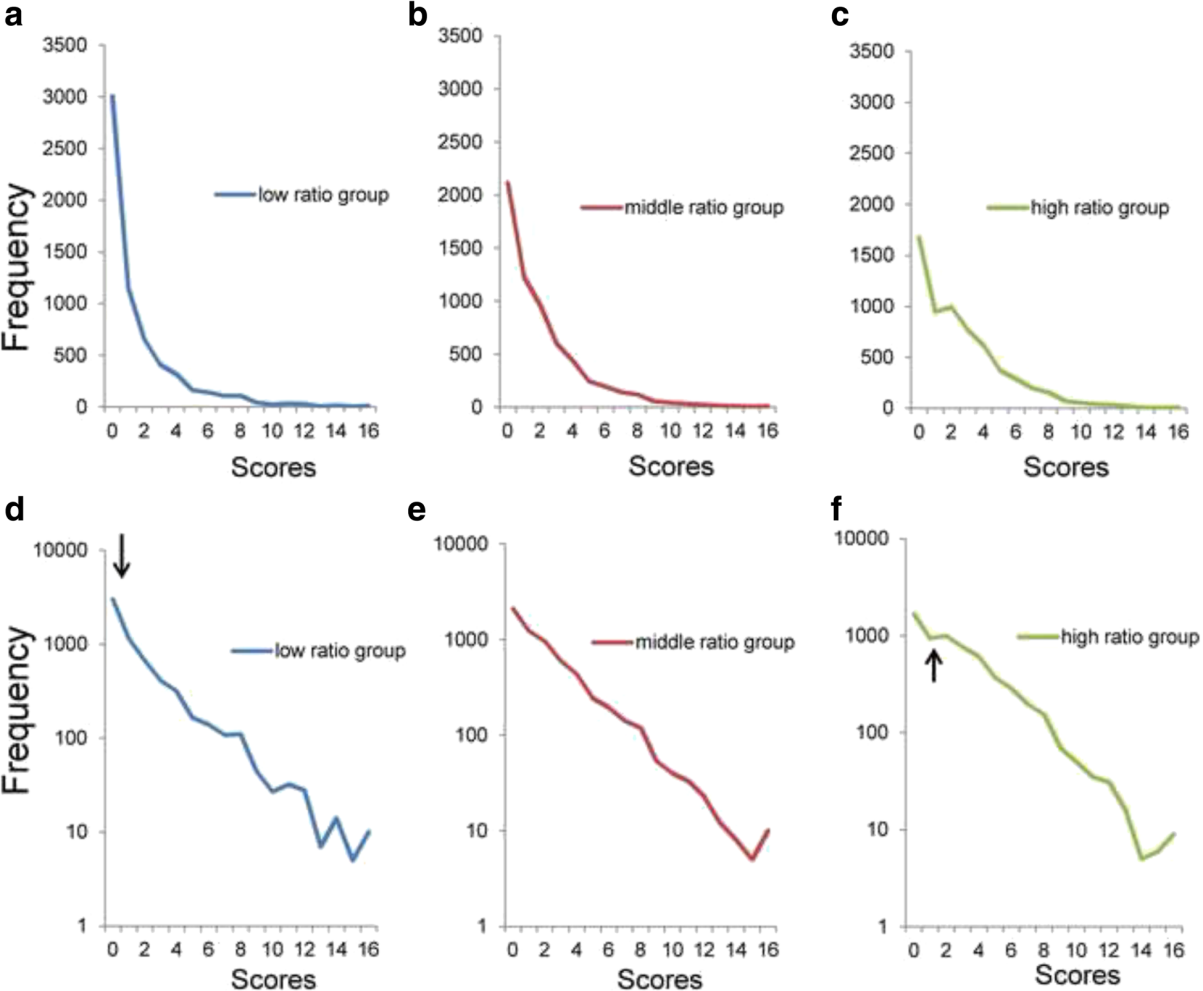
Distributions of the total scores of 4 items. **a** Low ratio group (items 1, 4, 5, and 6), (**b**) middle ratio group (items 1, 3, 4, and 5), and (**c**) high ratio group (items 1, 2, 3, and 5) on a normal scale. **d** Low ratio group, (**e**) middle ratio group, and (**f**) high ratio group on a semi-logarithmic scale. While all distributions of the three groups are right-skewed, the frequencies of the zero score differed between groups (**a**, **b**, and **c**). Using semi-logarithmic scales, all three groups showed linear patterns with similar gradients. At the lowest end of the scores, the distribution for the low ratio group (**d**) exhibited slightly higher frequencies than predicted from the linear pattern, while the distribution for the high ratio group (**f**) exhibited slightly lower frequencies than predicted from the linear pattern. The divergence of the actual data from the predicted linear pattern at the lower end of the distribution was not evident for the middle ratio group (**e**)

Figure 4 depicts the total score distributions for the three groups. While all three distributions were positively skewed, they differed in frequency of the zero score (Fig. 4a–c). Using a semi-logarithmic scale (Fig. 4d–f), all three groups exhibited linear patterns with similar slopes. As the arrows point, the distribution of the low ratio group (Fig. 4d) showed mildly higher frequencies than expected from the linear patterns, while the distribution of the high ratio group (Fig. 4f) showed mildly lower frequencies than expected from the linear patterns. An apparent gap between the frequencies at the lowest end of the total score and the linear pattern was not detected in the middle ratio group (Fig. 4e).

Exponential regression analysis was performed on data from the low ratio group (y = 2072e^-0.358x^, R^2^ = 0.95), middle ratio group (y = 2687e^-0.371x^, R^2^ = 0.98), and high ratio group (y = 3088e^-0.372x^, R^2^ = 0.97). The analysis revealed high R^2^ values in three distributions with similar parameters (−0.358 to −0.372).

### Distributions of the sum of 3 items

Three groups were chosen: low ratio group (items 1, 4, and 6), middle ratio group (items 1, 3, and 5), and high ratio group (items 2, 3, and 5). The low ratio group consisted of items from the first to the third rank order of the ratio of “a little” to “none,” the middle ratio group consisted of items from the third to fifth, and high ratio group consisted of items from the fourth to sixth. The averages of the ratio of “a little” to “none” were 0.20, 0.48, and 0.66 for the low ratio group, middle ratio group, and high ratio group, respectively.

**Fig. 5:**
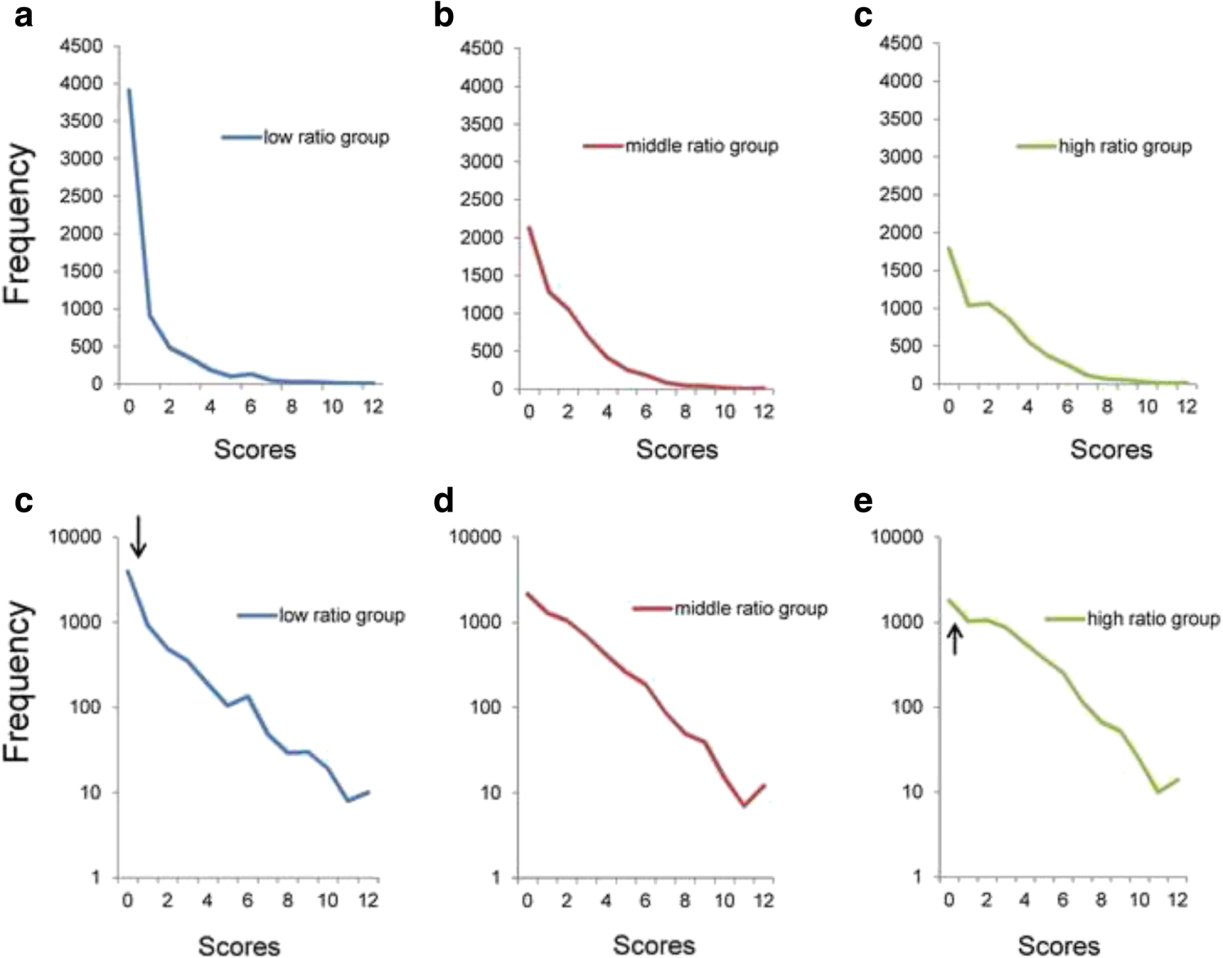
Distributions of the total scores of 3 items. **a** Low ratio group (items 1, 4, and 6), (**b**) middle ratio group (items 1, 3, and 5), and (**c**) high ratio group (items 2, 3, and 5) on a normal scale. **d** Low ratio group, (**e**) middle ratio group, and (**f**) high ratio group on a semi-logarithmic scale. While all distributions of the three groups were right-skewed, the frequencies of the zero score differed between groups (**a**, **b**, and **c**). Using semi-logarithmic scales, all three groups showed linear patterns with similar gradients. At the lowest end of the scores, the distribution for the low ratio group (**d**) exhibited higher frequencies than predicted from the linear pattern, while the distribution for the high ratio group (**f**) exhibited lower frequencies than predicted from the linear pattern. The divergence of the actual data from the predicted linear pattern at the lower end of the distribution was not evident for the middle ratio group (**e**)

Figure 5a–c depicts the distributions of the total scores for the three groups. While all three distributions were positively skewed, the high ratio group exhibited a plateau between points 1 and 2 (Fig. 5c). Using a semi-logarithmic scale (Fig. 5d–f), all three distributions exhibited linear patterns with similar slopes. Between points 0 to 2, the low ratio group (Fig. 5d) showed higher frequencies than expected from the linear pattern, while the high ratio group (Fig. 5f) showed lower frequencies than expected from the linear pattern. An apparent gap between the frequencies at the lowest end of the total score and the linear pattern was not detected in the middle ratio group (Fig. 5). At point 12, all three distributions showed higher frequencies than expected from the linear patterns.

Exponential regression analysis was performed on data from the low ratio group (y = 2559e^-0.463x^, R^2^ = 0.96), middle ratio group (y = 4158e^-0.484x^, R^2^ = 0.98), and low ratio group (y = 3942e^-0.447x^, R^2^ = 0.97). In line with semi-logarithmic scale findings, the regression analysis showed high R^2^ values in all three distributions with similar rate parameters (−0.447 to −0.484).

### Distributions of the sum of 2 items

Finally, to analyze the patterns of the sum of 2 item scores, three groups were chosen: low ratio group (items 4 and 6), middle ratio group (items 1 and 5), and low ratio group (items 2 and 3). The low ratio group consisted of the first and second items in the rank order of the ratio of “a little” to “none,” middle ratio group consisted of the third and fourth, and high ratio group consisted of the fifth and sixth. The averages of the ratio of “a little” to “none” were 0.15, 0.38, and 0.76 for the low ratio group, middle ratio group, and high ratio group, respectively.

**Fig. 6:**
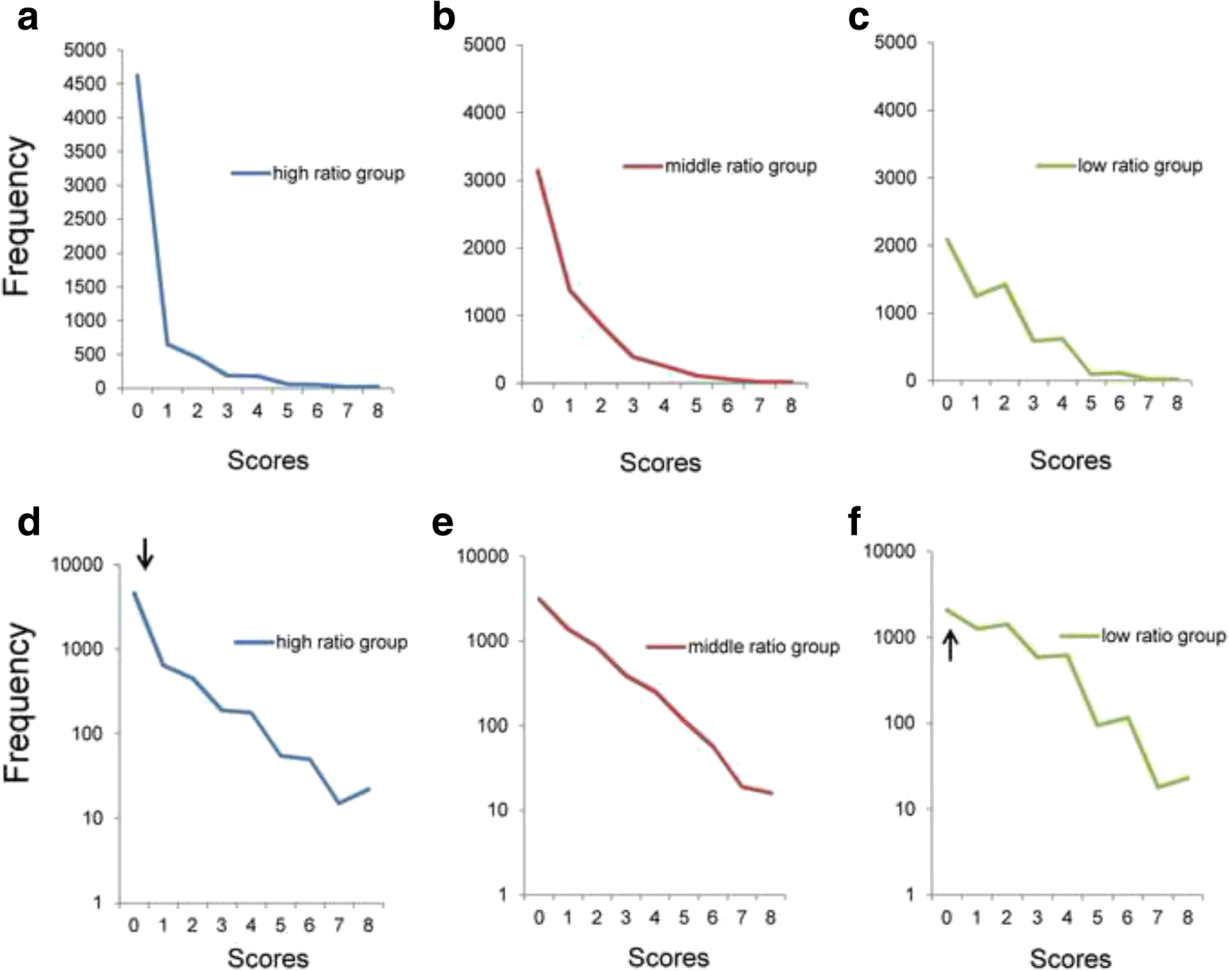
Distributions of the total scores of 2 items. **a** Low ratio group (items 4 and 6), (**b**) middle ratio group (items 1 and 5), and (**c**) high ratio group (items 2 and 3) on a normal scale. **d** Low ratio group, (**e**) middle ratio group, and (**f**) high ratio group on a semi-logarithmic scale. While all distributions of the three groups were right-skewed (**a**, **b**, and **c**), the high ratio group fluctuated between points 0 and 8 (**c**). Using a semi-logarithmic scale, while the middle ratio group showed apparent linear patterns (**e**), the low ratio group (**d**) and high ratio group (**f**) fluctuated considerably. The distribution for the low ratio group from points 0 to 1 exhibited higher frequencies than predicted from the linear pattern (**d**), while the distribution for the high ratio group exhibited lower frequencies than predicted from the linear pattern (**f**)

Figure 6a–c shows the three distributions of the sum of 2 item scores. While the three distributions were positively skewed, the high ratio group fluctuated between points 0 and 6 (Fig. 6c). Using a semi-logarithmic scale, while the middle ratio group (Fig. 6e) showed a linear pattern, the low ratio group (Fig. 6d) and high ratio group (Fig. 6f) fluctuated considerably. The distribution for the low ratio group (Fig. 6d) from points 0 to 1 showed higher frequencies than expected from the linear pattern, while the distribution for the high ratio group (Fig. 6f) showed lower frequencies than expected from the linear pattern.

Exponential regression analysis was performed on data from the low ratio group (y = 3674e^-0.638x^, R^2^ = 0.93), middle ratio group (y = 6132e^-0.677x^, R^2^ = 0.99), and high ratio group (y = 6051e^-0.627x^, R^2^ = 0.92). Although the regression analysis revealed a higher R^2^ value in the middle ratio group, the low ratio group and high ratio group did not exhibit higher R^2^ values compared to the middle ratio group, probably due to the fluctuations of the distributions. The estimated rate parameters were similar among the three groups (−0.627 to −0.677).

## Discussion

The present studies yielded three main findings. First, irrespective of the choice of items, the sum of item scores of K6 exhibited an exponential pattern,with the exception of the lower end of the distribution. Second, the estimated rate parameters of the regression analysis were similar among groups with the same number of items, and increased in proportion to the number of chosen items. Lastly, the ratio of “none” to “a little” of the chosen items contributed to the distributional pattern of the total scores at the lower end of the distribution.

Our results reveal that the sum of K6 item scores in various combinations shows an exponential curve, with the exception of the lower end of the distribution. These results concur with previous findings using the CES-D [20], suggesting that regardless of the depression screening scales, the sum of item scores approximate an exponential pattern, with the exception of the lowest scores. A theory proposed by our research group may explain why the sum of item scores for depression screening scales follow an exponential pattern [11, 24, 25]. Our theory consists of three main parts. First, all item scores are manifest variables, which correspond to a single latent variable. Second, the latent variable of all item scores follows an exponential distribution. Finally, the threshold of item scoring differs from individual to individual, and forms a frequency distribution according to the single latent variable [11, 24, 25]. In favor of this theory, our recent study simulating the three parts showed that the total score distribution exhibits the distributional pattern of a latent variable, with the exception of the lowest symptom counts [24].

A total score of 13 on the K6 scale has been used as a cut-off point for serious mental illness [26]. Figure 2 illustrates that exponential pattern extends almost the entire range of K6 scores beyond the cut-off point. These findings suggest that depressive symptoms are better conceptualized as a continuous dimension rather than a discrete category. Our findings concur with the results from the taxometric analysis, which is a statistical method specially designed to identify whether given constructs are categorical or dimensional [27, 28].

Based on the exponential patterns of the total and individual item scores, we propose that the single latent variable on depression screening scales follows an exponential distribution. On the other hand, parametric statistics and latent trait theory, which assume a latent variable to be a normal distribution, are widely used for the analysis of depressive symptoms [29, 30]. However, there has been no reliable evidence that depressive symptoms follow a normally distributed latent variable. The weakness with the normally distributed latent variable is that it does not explain why a normally distributed latent variable causes the distributional patterns presented in this and previous studies.

The finding that the total scores showed an exponential distribution potentially enables further insight into the mechanism of depressive symptomatology. Typically, an exponential distribution emerges when individual variability coexists with total stability, for example, energy levels within atoms and individual income [31]. Regarding individual variability and total stability, the mood of individuals often changes depending on the situation, and the total score distribution on depression screening scales in the general population is stable between the ages of 30 and 69 [11, 12, 32]. More research is required to explore the mechanism of depressive symptomatology.

The estimated rate parameters of the individual and total scores were −1.02 to −1.38, −0.63 to −0.68, −0.45 to −0.48, −0.36 to 0.37, −0.30 to −0.31, and −0.26 for each item response, and 2, 3, 4, 5, and 6 items, respectively. The estimated parameters increased in proportion to the number of chosen items, and the distributions of the same number of items showed similar parameters, consistent with previous reports using the CES-D [20].

The previous paper using the CES-D indicated that the estimated rate parameters of the distributions for 2, 4, 8, and 16 items were −0.62 to −0.93, −0.41 to −0.52, −0.26 to −0.29, and −0.14, respectively [20]. Interestingly, the rate parameters were similar between 6-item scores of the K6 (−0.26) and 8-item scores of the CES-D (−0.26 to −0.29), and between 3-item scores of the K6 (−0.45 to −0.48) and the 4-item score of the CES-D (−0.41 to −0.52). The maximum total scores for both 6-item scores of the K6 and 8-item scores of the CES-D have the same maximum total score of 24 points, and 3-item scores of the K6 and the 4-item score of the CES-D have the same highest possible score of 12 points. These results raise the possibility that the highest possible score of chosen items may contribute to the estimated rate parameter of the total score distributions of the chosen items. More research is required to understand the mechanism of the rate parameters.

Our findings demonstrate that, at the lower end of the distribution, the total scores of chosen items exhibited different patterns according to the ratio of “a little” to “none.” More specifically, the distributional pattern for the low ratio group with 2, 3, and 4 items showed higher frequencies than expected from the fitted exponential curve, whereas the high ratio group with 2, 3, and 4 items showed lower frequencies than expected from the exponential curve. The middle ratio group showed an exponential pattern for the entire range. Our results concur with previous reports using the CES-D [20]. Taken together, these findings support that the probability of “none” plays a vital role in predicting the pattern of total scores of the chosen item at the lowest scores.

Several analyses of CIS-R data from the British National Household Psychiatric Morbidity Survey have revealed that, although exponential regression analysis fitted total CIS-R scores, the actual frequencies at the lower end of the total CIS-R scores were higher compared to those expected from the regression analysis [10, 33, 34]. The CIS-R is scored with binary response options (absence or presence) [35], suggesting that the pattern at the lower end of the total CIS-R scores depends on the percentage of “absence.” Based on our analysis of the CIS-R data [36], the average percentage of “absence” in CIS-R data (90.1%) was much greater than the percentage of “none” in the present study (63.4%).

Our findings indicated that the total scores fluctuated more with the increase of total scores on a semi-logarithmic scale (Fig. 2b). These results are congruent with previous studies [11, 17], indicating that the small sample sizes resulted in fluctu0ation at the higher end of the distribution. In fact, as the total scores increased, the frequencies decreased.

The findings of this research need interpretation in light of some limitations. Although we demonstrated that an exponential curve fitted the total and individual item score data, we did not fit other functions to the data. Generally, the most important part of model selection is to identify patterns in data. However, there is little published data on the patterns of total and individual item scores. Thus, using histograms and regression analysis, we identified the patterns. More research is needed to assess the comparative fit of other patterns to the data from the Interuniversity Consortium for Political and Social Research (ICPSR) repository.

This research has some methodological strengths. First, as noted in the Introduction, data of MIDUS are large sample sizes with limited selection bias. Moreover, since all data are archived at the ICPSR repository and are publicly available, researchers can easily review the present findings using the raw data. Second, even though the method for this research was simple (visualizations with histograms), it enabled identification of a complex pattern of item responses; this could have been overlooked if item responses were not examined visually. Graphical analysis is indispensable in exploratory data analysis for complex models [37]. Finally, the results of this research provide insights into depressive symptomatology in the general population. It would be interesting to determine whether these findings can be generalized to clinician-rated depression scales.

## Conclusion

The findings from this research suggest that total scores on the depression screening scales follow an exponential pattern, with the exception of the lower end of the distribution. Based on the distributional patterns observed in this study, it is necessary to reconsider the statistical model of normally distributed depressive symptom scores, which is often presupposed in population studies on depressive symptomatology. The present results potentially enable further insight into the mechanism of depressive symptomatology, and estimation of how depressive symptoms distribute in a general population.

## Abbreviations

BRFSS: Behavioral Risk Factor Surveillance Survey
CDA: Confirmatory data analysis
CES-D: Center for Epidemiologic Studies Depression Scale
CIS-R: Revised Clinical Interview Schedule
EDA: Exploratory data analysis
K6: Kessler Screening Scale for Psychological Distress
MIDUS: National Survey of Midlife Development in the United States
NHIS: National Health Interview Survey
PHQ: Patient Health Questionnaire
RDD: Random-digit-dialing
